# Countdown to 2030: Adolescent sexual and reproductive health in Pakistan

**DOI:** 10.12669/pjms.41.12.13828

**Published:** 2025-12

**Authors:** Summia Khan

**Affiliations:** Dr. Summia Khan Azra Naheed Medical College, Superior University, Lahore, Pakistan. Email: ksummia@gmail.com

Adolescence is a transitional phase from childhood to adulthood, accompanied by physical as well as psychosocial changes. Health is a basic human right, and Sexual and reproductive health (SRH) is essential to adolescent well-being. According to the United National Population Development Fund, SRH is complete physical, mental and social wellbeing relevant to the reproductive system. This definition has a broader scope which encompasses accessible health services, appropriate information, education and counseling.^1^-^3^

The international conference on population and development in 1994, proved a turning point for adolescent sexual and reproductive health through framing educational and service needs as a top priority. However, it was not given due significance through Millennium development goals (MDGs). Sexual and Reproductive health (SRH) of adolescents has been highlighted through sustainable developmental goals (SDGs) 3 & 5. Goal 3.1 and 3.7 advocates reduced maternal mortality and universal access towards the SRH. Goal 5.2 and 5.3 endorse termination of violence against females and childhood marriages.^3^-^5^

Adolescents face a diverse set of challenges in terms of health, which include communicable, non-communicable diseases, injuries and substance abuse. In terms of SRH, important domains are childhood marriages, adolescence motherhood, high fecundity, unmet need for contraception, human immunodeficiency virus (HIV) infection, violence, menstrual hygiene and female genital mutilation.^1^,^4^

Since 1994, due to demographic transition, the adolescent population has been increased from 1.1 billion to approximately 163 million till now. Child marriage and adolescent pregnancy have declined globally. Owing to rapid population growth, the absolute number of girls getting married before 18 years of age has been increased. Approximately 12 million girls got married before 18 years of age, and 5% of girls got married even before 15 years of age. However, birth rate among adolescents has dropped by 34.4%.^1^

Adolescent pregnancy can result in numerous complications for mothers such as postpartum hemorrhage, postnatal infection, obstetric fistula, increased morbidity and mortality. Babies of adolescent mothers suffer from asphyxia, pre-term birth and low birth weight. Also, adolescent pregnancy can lead to school/college dropout, resulting in financial dependence. Globally, around 21% of adolescent girls are using modern contraceptives and unmet need is 23%. Unmet need leads towards unsafe abortions.^1^,^4^,^6^,^7^

As HIV was part of MDGs as well hence globally HIV incidence has declined 50% however, children received HIV through vertical transmission are adolescents now representing a 50% rise in existing HIV cases. Except chlamydia, all other sexual transmitted infections have been increased among adolescents. Risky sexual behavior such as unprotected sexual practices, multiple and concurrent intimate partners can lead to HIV infection. Sociodemographic profile including male gender, adolescent age, illicit drug usage, peer pressure and family attributes contribute towards risky sexual behavior.^1^,^4^,^8^ Reproductive tract cancer prevalence is quite low among adolescents, but Disability-adjusted life years (DALY) attribution has been increased for ovarian and breast cancer among females and testicular cancer among males. Menstrual hygiene and management is quite low among females due to cultural and taboo issues. Female genital mutilation has declined. There is a rise in violence, especially against females. Approximately 29% of adolescent women face violence from their intimate partner. These women are more predisposed to mental health issues such as anxiety, stress, and depression. Gynecological issues, chronic pain and sexually transmitted infection (STI) are also reported as a consequence of violence.^1^,^4^,^9^

Pakistan is one of the populous countries in the world and about 23% of the Pakistani population is adolescent. It has been reported that 50% of births occur among adolescent girls residing in rural areas. According to Pakistan Demographic & Health (PDHS) Survey 2017-18, the Adolescent birth rate was 46 per 1000. Approximately more than three fourths of women had unmet need for contraception. Due to high adolescent birth rate, there is increase in maternal and neonatal morbidity and mortality reported in Pakistan. Adolescent marriages have been reported in about 15% in girls and 3% in boys. Physical and sexual violence has been reported among 33% of adolescent women.^9^-^11^ Recent data regarding HIV incidence, STI (sexual transmitted infection), Menstrual hygiene and genital mutilation is not available.

For the 2030 targets of SDGs, the countdown has begun, and the clock is ticking. International agencies are calling for urgent action. Based on research and recent data, it’s the right time to initiate intervention which can directly bring change in adolescent health. A multi-sectorial approach with interventions at health services delivery point, educational institutes, personal level, youth centers and community mobilization should be carried out. In the USA, “Voices for Health Kids” initiative brought change through an advocacy campaign and community mobilization. In Malaysia, through the “HPV school initiative”, the government collaborated with pharmaceutical companies, media and parents. Anemia was prevented through a school based initiative in Indonesia. In South Africa, adolescent girls were empowered through the “She Conquers” campaign. As far as Pakistan is concerned, youth empowerment is limited to research and a few donor funded programs only. The “Sihaat Mand Khaandaan (SMK) Project: Health Families for Pakistan Through Accelerating Sexual Reproductive Health (SRH) and Family Planning (FP) Services” was initiated in Gilgit Baltistan funded by the Government of Canada and assistance by “the Aga Khan Rural Support Program (AKRSP)”. Under this program, the “Adolescent Friendly Spaces (AFSs)” initiative was implemented. Now about 17 spaces have been established to provide Sexual and reproductive health services, family planning, Life Skills Based Education (LSBE) along with formal education & computer training.^12^,^13^

Pakistan has one of the largest population dividends in the form of its adolescent population. Sexual and reproductive health is the most neglected aspect for teens here due to socio-cultural norms. In a digital era, where cultural norms are being challenged worldwide; it is the right time to match the momentum of global improvements in adolescent SRH. It can be achieved by placing adolescent health as top agenda with allocation of funds. There is a dire need for extensive research in this domain for evidence-based investment. Moreover, health service delivery to facilitate adolescents must be ensured. Technological solutions can be utilized to improve the accessibility of services and interventions. Despite gender disparities, adolescent SRH should be provided to marginalized boys in a broader context.
